# Objects as Communicative Mediators in Children With Autism Spectrum Disorder

**DOI:** 10.3389/fpsyg.2020.01269

**Published:** 2020-06-17

**Authors:** Federico Manzi, Giulia Savarese, Monica Mollo, Antonio Iannaccone

**Affiliations:** ^1^Research Unit on Theory of Mind, Department of Psychology, Università Cattolica del Sacro Cuore, Milan, Italy; ^2^Department of Medicine, Surgery and Dentistry, University of Salerno, Salerno, Italy; ^3^Department of Human, Philosophical, and Education Sciences, University of Salerno, Salerno, Italy; ^4^Institut de Psychologie et Éducation, Université de Neuchâtel, Neuchâtel, Switzerland

**Keywords:** autistic children, socio-material perspective, object use, communicative mediators, children play

## Abstract

In recent years, the socio-material perspective has informed an important interdisciplinary debate concerning the role of the physical world (i.e., the objects) in human psychological development. Several studies in the field of developmental psychology showed positive achievements in explaining the relationship between the subject and the social context through a socio-material approach, in particular in the early development. The importance of objects was also recognized in children with autism spectrum disorder (ASD), showing that these children are characterized by alterations in the use of the objects from early development. Some studies highlighted that objects could be a facilitator in the interactions between children with ASD and peers. However, the role of objects was not sufficiently investigated in interactions between children with ASD and adults. The main purpose of the present study was to investigate in children with ASD the communicative function that the activities with objects assume in the interactions with adults, highlighting the mediator role of objects in these interactions. More generally, this study also aims to highlight the relevance of adopting a socio-material perspective to explore some neglected aspects of the psychological activity of children with ASD. To test this hypothesis, we conducted an extensive exploratory study, collecting data from a sample of 3-year-old (*N* = 18; *F* = 3) and 4-year-old (*N* = 26; *F* = 3) with ASD. Children were observed in a free-play situation with an adult. They were free to choose an object from a predefined set. Through quantitative data, we have described the general characteristics of the manipulation of objects; through qualitative data, we aimed to capture and describe, in microgenetic sequences, some characteristics of children’s activities, defined as socio-material. The analysis of the socio-material activities suggested the role of objects as mediator of the interactions between children with ASD and adults.

## Theoretical Frame

The socio-material perspective emphasizes the role of both social and material dimensions of artifacts – conceived as closely interrelated – in psychological activity and investigates which features of artifacts can affect children’s social interaction patterns. Several studies have highlighted the role of objects in the very early stages of psychological development, both in allowing the expansion of psychological activity and as regulators of communication between social partners (Iannaccone, 2015; Moreno-Núñez et al., 2017; Manzi, 2018; Cattaruzza, 2019; Cattaruzza et al., 2019a,b).

The field of investigation of the role of objects in psychology is very broad and informed by the contributions of various theoretical perspectives and empirical approaches. Among others, Piaget, Vygotsky and Moscovici have tried to understand in very intriguing ways the role of objects in human development. In his work, Piaget (1928, 1952a, 1954, 1962, 1972) emphasizes how children actively build their own cognitive worlds, organizing knowledge into patterns, or mental structures, that serve to represent the reality to which they must adapt. Piaget’s approach underlines the interaction between the individual and their environment, without clearly overcoming the subject–object dualism, as the socio-material approach advocates:

*A child sometimes sucks his thumb as early as the second month, grabs objects at around 4–5 months, then shakes them, swings them, rubs them and finally learns to throw them and catch them. These behaviors presuppose at least two poles: on the one hand is accommodation, since it is necessary to adjust movements and perceptions to the objects themselves, and on the other hand is assimilation of objects to one’s own activity, since the child is not interested in the object in itself, but in so far as it can serve as “food” for a previous behavior or in the process of being acquired. This assimilation of the reality with sensory-motor schemes presents itself in two complementary aspects: it is an active repetition and consolidation (hence the “circular reaction” described by Baldwin), (…) it is “mental digestion,” that is to say perception or conception of the object according to its incorporation into a real or possible action: (…) In this regard, it is obvious that this double function of assimilation is only one in concrete activity, because it is to the extent that the subject repeats his behaviors by reproductive assimilation that he assimilates objects to actions and that these become by this very fact patterns. These schemas then constitute the functional equivalent of concepts and subsequent logical relations.* (Piaget, 1952b, p. 15, par. 5).

However, despite the dualistic position, Piaget’s works made essential contributions to understand the relationship between cognitive development and physical reality. For example, the micro-genetic method has allowed for comprehending how we can provoke and observe the transformations of this relationship between the subject and the physical world in a limited space–time sequence. This is a fundamental methodological condition for understanding the development of materiality in child development.

Vygotsky (2004), while sharing Piaget’s view of the child as an active builder of their own knowledge, highlights that cognitive development largely depends on the progressive appropriation of psychological tools made available by society and produced by culture (Ben-Ari and Kedem-Friedrich, 2000). Moreover, he argues that the transition from practical intelligence, interrelated to reality, to a more abstract intelligence (shared and sharable) is mediated by cultural artifacts, mainly language. Vygotskjian’s approach conceives artifacts as symbolic mediators (both material and psychological) of human social cognition and as products of historical and cultural development. These are used by humans to interact with others and reality, and to reflect on their activities. In this sense, objects, as artifacts (and largely socio-material ones), represent a constitutive component of human life on two different levels: at an interpersonal level, these “tools” regulate communication, interaction and all social activities; at an intrapersonal level, they extend human functions and, once internalized, guide thought (Moro, 2011). In these terms, the psychological activity with and the awareness of artifacts depend on the nature of the interaction between the human and non-human components of this dialectical dyad. As well-explained by Vygotsky (see quote below), a child’s play, involving both above-mentioned components, is a situation that allows them to explore the physical and social world (Iannaccone et al., 2019). In this perspective, a child’s play does not represent a simple reproduction of the experience but a creative reworking of it:

A child’s play very often is just an echo of what he saw and heard adults do; nevertheless, these elements of his previous experience are never merely reproduced in play in exactly the way they occurred in reality. A child’s play is not simply a reproduction of what he has experienced, but a creative reworking of the impressions he has acquired. He combines them and uses them to construct a new reality, one that conforms to his own needs and desires. Children’s desire to draw and make up stories are other examples of exactly this same type of imagination and play. (Vygotskij, 1976, p. 11).

Also, social psychology has provided some interesting contributions to conceptualizing the notion of objects and especially the mediating role that the partner assumes in the interaction. In this regard, Moscovici (1976), assuming a broad psychosocial perspective, adopts a triadic model. The key aspect of Moscovici’s theory is represented by a ternary reading of facts and relations, replacing the typical binary approach of the two terms of subject and object with one of three terms: the individual subject (I), the social subject (Other) and the Object. The I–Object relationship is always mediated by the Other; it can take a static form as “co-presence” or a dynamic form as “interaction,” and can lead to changes in thinking and behavior. Although this approach assumes a continuous interchange between the three elements of the triadic relationship, the dialogical theories provide an important enhancement to the understanding of materiality. Linell (2009), adopting a dialogical approach, considers human interactions impossible without the presence of objects, considered as artifacts that embody the cultural heritage of the human species (see also Tomasello, 2016). In fact, as Linell (2009) states, “many forms of human cognition and communication cannot occur without artifacts” (p. 345). From the dialogical approach, human interactions are characterized by combined actions during which humans using objects to transform their perspectives of the interactive world (Linell, 2009).

According to this point of view, mental activity cannot be considered as a “decontextualized” activity “in solitude” but it largely depends on the continuous interaction of individuals with the physical and social world (Clark and Chalmers, 1998). The process of thinking can be considered as a social co-construction of the meaning of the social experiences that continuously involve humans (Perret-Clermont, 2004; Iannaccone and Bruner, 2010; Iannaccone et al., 2016). Thus, thinking is a “form of social practice” (Radford, 2003) that refers to lived experience in concrete situations. Based on these assumptions, thinking activities are triggered by actions in a specific socio-material activity context: these activities individually, isolated from their contexts, have no meaning (Zucchermaglio, 1996; Ligorio, 2010; Coppola et al., 2019). The above considerations allow for defining the main assumption of the socio-material perspective: human development is intrinsically bound to the material components of the social context.

The attention to the role of objects in children’s social interactions is also addressed in the research field of developmental psychology. Pioneering studies have shown that objects can support children’s interactions in early development (Jacobson, 1981; De Stefano and Muller, 1982; Lieber and Beckman, 1991) and stimulate more complex interactions among children (De Stefano and Muller, 1982). The importance of objects in children was also observed in interactions between children and adults, in which the children’s use of objects changes as a function of the sociocultural background of the caregivers, who transmit this heritage (Bakeman et al., 1990; Tomasello et al., 1990). Actually, the role of objects in children’s interactions—both with peers and adults—is even more important considering also the recent design of new relational artifacts (Turkle, 2004), i.e., robots (Manzi et al., 2017, 2020; Marchetti et al., 2018, 2020; Di Dio et al., 2019, 2020a,b; Manzi et al., 2020). Recently, several studies have highlighted the role (and uses) of objects, as mediators, in the adult–child interactions in early development (Rodríguez and Moro, 1998; Dimitrova and Moro, 2013; Rodríguez et al., 2015; Moreno-Núñez et al., 2017). In these interactions, adults acquire the role of the scaffolder, teaching the child the different (conventional) uses of the object (Moro, 2011, 2014). In Vygotskian terms, adult–child activities are part of a process of the co-construction of knowledge that involves a negotiation of intersubjective meanings. In this sense, the object represents a support in the interactions between child and adult, becoming one of the components of secondary intersubjectivity (Trevarthen and Hubley, 1978). This leads to a decisive transformation of the children’s interaction: from a dyadic interaction (child–object or child–adult) to a triadic interaction (adult–child–object). In typical development, it is clear how the use of objects represents a crucial element of interactions (Barthélémy-Musso et al., 2013; Rodríguez et al., 2015).

### The Object in Autism Spectrum Disorder

In the previous paragraph, we delineated some fundamental theoretical coordinates to define the importance that objects have in children’s social interactions, particularly in typical development. In the present paragraph, we will briefly outline the importance of studying the role of objects for children with autism spectrum disorder (ASD). As described by DSM 5, autism is a persistent deficit in communication and social interaction that manifests itself in various contexts (DSM, 5). Additionally, autism is characterized by restricted and/or repetitive behavior patterns, interests or activities: in this sense, children’s modalities of the use of objects represent an important element to be considered in the diagnosis. It is not intended here to analyze the children’s use of objects as a diagnostic factor, but only to highlight that the objects represent an element of diagnostic interest. With respect to the use of objects, Kanner (1943) was among the first to note that, despite differences and limitations, children with ASD exhibit a particular interest in objects. Generally, children with ASD present altered patterns of object exploration and manipulation starting from an early stage of development (Sigman and Ungerer, 1984; Bruckner and Yoder, 2007; Mottron et al., 2007; Ozonoff et al., 2008; for a review see Williams et al., 1999). Furthermore, several pieces of research have shown alterations related to the conventional use of objects, namely the appropriate use of everyday objects (Lord, 1983; Loveland and Tunali, 1991; Bachevalier, 1994; Williams et al., 2005). In addition, the use of objects in children with ASD has been extensively studied in functional and symbolic play, showing alterations to use them also in play (Jarrold et al., 1993; Jarrold, 2003). However, other studies have identified how objects can become mediators of interactions between children with ASD and peers (Romanczyk and Goren, 1975; Lord, 1983, 1984; Lord and Hopkins, 1986). Thus, the objects with a communicative function in the interactions between children with ASD and peers has been recognized in literature, although not further investigated. However, no study has ever specifically analyzed 310 the role of objects as mediators of interactions between children with ASD and adults in a socio-material perspective.

### Aims

The main purpose of the present study was to investigate in children with ASD the communicative function that the activities with objects assume in the interactions with adults, highlighting the mediator role of objects in these interactions. To fulfill this aim, we implemented a quasi-experimental design, observing different forms of children’s “playful” interactions. A broader aim was to provide insights in adopting a socio-material approach to the activities of children with ASD analyzing the wider context.

## Materials and Methods

### Participants

Forty-four (44) Italian preschool-age children with ASD participated in the experiment. The children were divided into two age groups as follows: 3-year-olds (*N* = 18, *F* = 7; *M* = 32.94, *SE* = 4.13) and 4-year-olds (*N* = 26, *F* = 10; *M* = 48.36, *SE* = 5.42). The children were recruited from different rehabilitation centers of the Campania region, Italy. Inclusion criteria for the two groups are related to the child’s diagnosis of ASD, according to the *Diagnostic and Statistical Manual of Mental Disorders* criteria (DSM 5: American Psychiatric Association [APA], 2013) made by experts. The children’s parents received a written explanation of the procedure of the study, the measurement items and the materials used, and they gave written consent. The number of participants correspond with the number of children recruited.

### Measures

#### Socio-Material Use of Objects Checklist

The “Socio-Material Use of Objects” checklist (SMUO; Savarese et al., 2017; Iannaccone et al., 2018) consists of 14 items (see Appendix 1). The items explore both the social behaviors of the child toward an interactive partner and the activities displayed toward the objects. Thus, the checklist focuses on how the child “behaves” with the object within an interaction with a partner. For each item, the observer assigned score 1 when the behavior or activity occurred. The sum of the 14 items is grouped into a factor named *Social Modalities*, which ranges from 0 to 14. This score allows for estimating children’s social interaction patterns in a context involving an object and a partner. In addition, the observer has to fill an observational section to provide detailed information concerning the events occurring during the play session.

According to the socio-material perspective and the scientific literature (Dominguez et al., 2006; Bruckner and Yoder, 2007), children’s activities with objects were classified according to three criteria: (1) *Sensory-Motor Activities* (SMAs), typical of interactions in which the child uses the toy as a means to engage in a sensorial experience (involving touch, hearing, sight, smell and/or taste), including any self-stimulating behavior with repetition of gestures or specific uses of an object (stacking, piling or slamming to hear a noise); (2) *Canonical Activities* (Cas), referring to using the functional characteristics of objects and uses encoded in the child’s past experience; (3) *Social-Interactive Activities* (SIAs), referring to the child that uses the object as a mediator tool that promotes the relationship with the adult.

### Procedure

The study involved two experimenters (the observers) and one experienced educator (i.e., the adult) who were qualified to work with children with ASD and to observe them in different interactive contexts. Two observers were involved for each child, allowing for a comparison between the two sets of observations.

The experienced educator introduced the children to a set of objects (toy cars, dolls, plasticine, cubes etc.) and they were free to choose their preferred object during the interaction. The materials were selected considering recommendations from previous studies showing potential preferences of the types of objects by children with ASD (Williams et al., 1999; Ziviani et al., 2001; Dominguez et al., 2006). Specifically, children could choose from objects that potentially elicited different types of play behavior (e.g., sensorimotor, canonical, symbolic). Both experimenters completed the checklist, verifying the presence of social behavior toward the adult and activities with the object during the play session. In addition, the two experimenters had to independently note what had occurred during the play session. The observational comments were also enriched with the considerations of the experienced educator at the end of each session. The observation of the free play session lasted about 10 minutes and was carried out in quiet rooms in different motor rehabilitation centers in the region of Campania, Italy.

## Results

### Analysis of Children’s Social Modalities

In addition to the diagnosis of autism provided by experts, SMUO has been used to assess both the social behavior exhibited by children toward their partner and their exploratory behavior toward the objects. The checklist allowed for a general score of the children’s social interaction modalities.

The ANOVA analysis of the mean score of *Social Modalities* of interaction shows a significant difference between 3- and 4-year-old children (3 years: *N* = 18, *M* = 1.11, *SE* = 0.75; 4 years: *N* = 26, *M* = 1.85, *SE* = 1.05, *p* < 0.05). Compared to the 3-year-old children, the 4-year-olds had a higher mean score and exhibited more social behaviors. Although this data seems to indicate an effect of age on children’s modalities of interactions, this score is not a diagnostic index of the severity of the pathology, so it is purely informative with respect to our sample of a greater presence of interactive behaviors in the older children’s group.

### Analysis of Children’s Activities With the Objects

As mentioned above, children’s activities were classified in three different type of activities (*SMAs*, *Cas* and *SIAs*) (for details see section “Measures”). Two independent judges evaluated the children’s activities. The inter-rater reliability scores were substantial (Cronbach’s Alpha = 0.71).

A Chi-square analysis (Table 1) did not reveal any differences in the type of activities between the 3- and 4-year-old children. The frequencies of the three categories observed refer to the number of participants. However, the 3-year-old children had a greater tendency to engage in *SMAs* (61.1%), while the 4-year-old children tended to engage in *SIAs* (34.6%) and *SMAs* (42.3%). The *Cas* frequencies were similar for both the 3- and 4-year-old children. Although children are of different ages, both groups (including the older ones) have typical ASD difficulties in the activities with objects. However, there is an increase in activities involving the Other as a function of age, although this increase is not significant.

**TABLE 1:** Distribution of activity types for 3- and 4-year-old children.

	**3 years old N (%)**	**4 years old N (%)**	**3 years vs. 4 years sign (*p)***
**SMAs**	11 (61.1)	11 (42.3)	ns
**CAs**	5 (27.8)	6 (23.1)	ns
**SIAs**	2 (11.1)	9 (34.6)	ns

*SMAs, *Sensory-Motor Activities;* CAs, *Canonical Activities;* SIAs, *Socio-Interactive Activities*.*

### Qualitative Data: Observations of the Children’s Activities

Qualitative data were obtained from microgenetic observations of children’s activities aiming at identifying the occurrence of behaviors that indicated the type of activity that the child performed with the object within the context of playful interactions with the objects. Specifically, the aim of the analysis of the qualitative data was to search for evidence on the use of objects as mediators in the social interaction between ASD children and adults.

Qualitative observations will be presented to provide examples of the different types of activities (*Sensory-Motor Activities*, *Canonical Activities*, and *Social-Interactive Activities*) in the play context. As mentioned above, in each scenario, the child freely chose the object and interacted with the adult. All of the observations were conducted in May 2019.

#### Sensory-Motor Activities

In *Sensory-Motor Activities*, the child uses the toy as a means to *activate* sensory channels (touch, hearing, sight, smell, taste) and for self-stimulation through the repetition of gestures and methods of using the object (stacking, piling, slamming to hear the noise, etc.).

Observation 1 – 22.02.2017 – Giovanni (*32-month-old male child*).

“[Giovanni] *spontaneously took the object and brought it toward his face, exploring it with his sense of smell. He then continued the exploration by manipulating the object for 30 s, after which he placed it on the ground.*”

The educator’s commentary underlines an activity based on sensoriality, whereby the child explores the object’s material characteristics.

“[Giovanni] *was then asked to take the object.* Giovanni *walked away from the operator and started walking around the room for about 30 s. He then returned to the activity, spontaneously taking the buildings, exploring them, and resting them on the floor. The operator started to construct a tower and the child imitated his action.*”

Here, the educator’s commentary highlights how the child does not respond to requests to take the object and share it.

In this interesting case, the child does not seem able to respond explicitly to the adult’s requests for interaction, and does not manifest linguistic behaviors of sharing. Nevertheless, the educator’s action of building a tower gives the child the opportunity to start an imitative action. In one sense, construction constitutes the socio-material element of the situation, allowing the child to share the realization of the task (albeit limited to the “remote” coordination of actions required by imitative conduct) and to somehow mediate the communicative function with the adult.

Observation 2 – 23.02.2017 – *Luigi (48-month-old male child).*

Luigi chose as an object a series of cubes made of a hard plastic material and with a concave space on one of its faces.

“*Luigi spontaneously begins to share attention with the aim of reaching the object, which is beyond his reach. He plays properly only with large construction cubes, which he manages to stack on a model.*”

A profile is outlined in which Luigi manages to make imitations but does not present shared attention or pointing.

“[When left to play with the cubes, Luigi] *shows an absorbent interest in the part underneath the cubes that is concave and where he usually puts his fingers […] the actions carried out by Luigi with the cubes include scattering, heaping, putting in a row, or overlapping. If stressed (about three times), the child returns to stack.*”

This observation indicates the child’s exploration of the object’s material characteristics, focussing only on the concave part of the cubes.

In the observation of Giovanni, it is interesting to observe how the object’s characteristics constitute real affordances that invite the child to perform specific sequences of actions, the nature of which obviously depends on the degree of psychological development and the severity of the autistic pathology. In the observation of Luigi, the interaction with the object constitutes an interesting element that highlights the child’s ability to act.

Observation 3 – 24.02.2017 – Francesco *(60-month-old male child).*

“[Francesco] *sniffs and visually chases soft rubber balls while sliding down… he has several balls available but chooses to always use the same ball.*”

The educator’s observation underlines the particular sensorial interests shown by the child. It is noteworthy that attempts to involve the operator or others in the game are absent.

Observation 4 – 25.02.2017 – Cosimo *(48-month-old male child).*

The toy chosen by Cosimo is an action figure. During the 120 s of observation:

“[Cosimo] *grabs the doll, shakes it, puts it in his mouth and then places it down, picks it up and shakes it again by rotating it in his hand, first to the right and then to the left, and walks simultaneously around the room with fast movements. He removes the hat from the doll’s head and returns to shaking only the hat first and then the rest of the toy with both hands. He puts the hat on the ground and shakes only the rest of the doll, gets up and sits down immediately afterward, takes other toys similar to the one in his hands, disassembles them and takes only the hat… in his hands he has two hats of two dolls, and he puts them in a row and looks at them.*”

The mouth, hands and eyes are the sensory channels that orientate Cosimo in his use of the toys, together with the repetition of some movements such as shaking the toys, their positioning in space and the noise they make.

“*Cosimo puts the dolls inside the container while shaking it to make a loud noise.*”

Both observing educators report the absence of attempts by the child to involve others in the game. Their accounts describe another interesting aspect of the use of the object that can provide interesting information about their cognitive activity. The object manipulated by Cosimo is a complex object, consisting of multiple elements. The observed manipulation shows how the child takes this complexity into account and lets his actions be guided by the object’s physical characteristics. Of course, it is impossible to deduce from the observational data the psychodynamic elements inducing the child to disassemble and reassemble the doll. These aspects, which are also of great interest in understanding the child’s psychological life, should be interpreted using paradigms of affective psychology.

#### Canonical Activities

Children engaging in *Canonical Activities* use the toy according to its extrinsic functions and insert it into the context of external reality.

Observation 5 – 26.02.2017 – *Carlo (36-month-old male child).*

Carlo chose a toy car made of hard plastic and featuring wheels that turn.

“*Carlo has a good understanding of the object of observation. In fact, he uses the toy car on the slide, looking at how it moves. The child’s observation of the interaction with the object lasts 5 minutes but he presents little eye-contact.*”

The educator’s comments highlight how the child can use the combination of two objects and understand their canonical function.

“*Some difficulties were encountered with regard to shared attention and the return of the object when requested by the adult. However, Carlo has good imitative skills; in fact, he imitates the movements of the adult when he places the car on the slide.*”

We can see that the child has well-established imitative abilities, even if explicit social conduct is not observed.

Observation 6 – 27.02.2017 – *Enzo (48-month-old male child).*

Enzo chose an electric piano made of hard plastic and composed of several keys.

“*Enzo took several minutes to examine the function of the various keys and imitated the behavior of the educator by switching [the piano] on and off several times during use. He pressed the keys of the piano only for a short period of time; instead, he preferred to listen to the output of the pre-recorded music and press the different keys to change the melody and volume.*”

These comments show that the child understands the canonical use of the electric piano, specifically how to make it produce sounds.

The notion of canonical manipulation allows us to ascertain that both Carlo and Enzo have acquired important social knowledge, at least as regards understanding the rules for the use of the object. The differences from uniquely sensory-motor activities are evident and allow for a more precise assessment of the methods the children adopt to relate to the social and material reality.

Observation 7 – 28.02.2017 – *Roberto (64-month-old male child).*

Roberto chose a soft wolf-shaped toy.

“*Roberto puts the stuffed animal in a seated position, then takes the food and puts it in the saucepan and mixes it with a spoon. He brings the food to the wolf’s mouth to feed it and then takes the food and drinks to bring them first to his own mouth and then toward the stuffed animal’s mouth. He imitates the non-verbal signs of drinking and sleeping.*”

The educator’s comments reveal a functional profile of Roberto with respect to his play. During the observation, Roberto showed his ability to combine the objects based on their specific configuration (he seated the wolf and put the kitchen tools back in their place) and based on their conventional characteristics (he brought the food closer to his mouth and to the wolf’s mouth to imitate the gesture of feeding). However, the child did not directly look into the other’s eyes while using the toys or when taking the objects.

Observation 8 – 01.03.2017 – *Loretta (48-month-old female child).*

The educator suggested that Loretta could play with a doll:

“*Loretta takes a doll and says, “Look how beautiful this doll is! She has very beautiful hair… do we comb it?” Loretta responds to the educator’s request by taking the doll, looking at it and caressing it. Then Loretta takes the comb from the ground, looks at it, touches it with her other hand, takes the doll’s hat off and combs its hair. Then Loretta takes the comb from the ground and combs the doll’s hair.*”

In this example, the child shows functional behavior toward the object and contextualizes the use of the doll and the objects at her disposal. She also demonstrates good imitation skills in using the object.

“After the educator demonstrates, Loretta begins to imitate the movements of the comb on the hair or to repeat, ‘This hair is beautiful, it is a beautiful color’.”

The cases of Roberto and Loretta offer clear examples of conduct that recognize the “canonical use” of objects, but the observed activities appear more advanced than in the previous cases in terms of interactive skills. In the observation of Roberto, the elements of the playful scene communicate with each other and social behaviors arise. In the observation of Loretta, there is even some element of direct communication with the educator accompanying the canonical manipulation of the object.

#### Social-Interactive Activities

In *Social-Interactive Activities*, the child explicitly uses the toy to enter a relationship with the adult.

Observation 9 – 02.03.2017 – *Melvis (36-month-old male child).*

Melvis chose a toy phone made of hard plastic and featuring four wheels.

“*Melvis moves the toy phone on its wheels; he takes the handset and passes it from one hand to the other, then he brings the phone to his ear and says “Pompo” (hello). Once the educator takes the phone and says “Hello,” Melvis does the same, looking him in the eyes for a few (two) seconds.*”

In this sequence of actions, we can observe how the child carries out an interactive activity involving the other by using an object, even if only for a short time.

Observation 10 – 03.03.2017 – *Stefano (44-month-old male child).*

Stefano chose a toy truck made of hard plastic and featuring four wheels.

“*Stefano pays attention to every part of the truck; he does not use the truck making repetitive movements. Stefano manipulates the truck in order to make movements related to its function (he puts a toy child in the driver’s seat, attaches the trailer and pushes it). Stefano holds the truck for one minute and 30 s. Stefano points and says the name of the desired object in order to get it.*”

In this interaction sequence, the child clearly organizes an activity coherent with the functional opportunities offered by the object. At the same time, the child needs basic interaction with the educator to reach his goals. The object’s required accessories propel the child to interact with the adult.

Observation 11 – 04.03.2017 – *Federico (60-month-old male child).*

Federico chose a ball as his game object.

“Federico looks at the ball in the basket and makes eye-contact with the therapist. He points at the object and says, ‘Do we play ball?’ Then the therapist asks, ‘Who is the goalkeeper?’ and Federico answers, ‘You are the goalkeeper!”’

Federico then kicked the ball several times and the therapist acted as the goalkeeper. This example shows the child employing an object (the ball) to involve the educator in the game.

## Discussion

Starting from a previous exploratory study (Iannaccone et al., 2018), this research aimed to deepen the understanding of the socio-material contexts and, in particular, the role of objects in the psychological functioning of children with ASD. In the present study, children with ASD aged three and four were observed within a situation of free play with an object freely chosen by the child from a predefined set of objects. The child was free to include the adult or not within its activity with the object. A general result concerned the positive effects of also adopting a sociomaterial perspective to the analysis of the interactions between children with ASD and adults. Furthermore, the results showed that children preferred sensory and motor activities with objects independent of age and that older children had more sophisticated modalities of interaction than younger children. Finally, another fundamental result emerged from the analysis of the observations of the child-adult interaction: objects can be useful mediators of interaction with adults.

With respect to the more general theoretical outcome, in recent years, the socio-material perspective has informed an important interdisciplinary debate concerning the role of the physical world (i.e., the objects) in human psychological development, involving different branches of psychology (Malafouris, 2013, 2019). This perspective, also introduced in educational psychology studies, has provided an opportunity to highlight the importance of analyzing the socio-material context in which the relationships occur, including the educational ones (Iannaccone, 2015, 2017; Cattaruzza, 2018; Cattaruzza et al., 2019a, b; Iannaccone et al., 2019). This study also promotes researchers’ awareness of the opportunities offered by this approach identifying the human mind and its development as an embodied, extended and distributed activity (Clark and Chalmers, 1998). Several studies in the field of developmental psychology showed positive achievements in explaining the relationship between the subject and the social context through a socio-material approach, in particular in the early development (Moro, 2011, 2014; Dimitrova and Moro, 2013; Rodríguez et al., 2015; Moreno-Núñez et al., 2017). These studies allowed for hypothesizing the adoption of the socio-material perspective in research and interventions with different pathologies and mental disorders, specifically with autism. This hypothesis arises from research that highlights how autism is characterized not only by a primary alteration in social relations but also by an alteration within the wider socio-material context. From these premises, our study provides for the first time the possibility to extend the socio-material approach, until now mainly used to explain typical development, even to atypical development. Specifically, our results concerning the objects as mediators of the relationship show that children with ASD actively use the sociomaterial context -albeit as a function of their symptomatic characteristics- to understand and explore the material and social world.

The results concerning the type of sociomaterial activity (Sensory-Motor Activities, Canonical Activities and Socio-Interactive Activities) that the children with ASD preferred in the experimental situation of our study allowed for being aware of the intricate interplay between the psychological and material components in the experiences of these children. The observations of the socio-material activities of these children with objects seem to lead to a non-linear interpretation of the development of different interactional modalities established between children and objects. This result is in line with previous studies showing that children with ASD present alterations in the use of the objects from early development (Williams et al., 1999; Ozonoff et al., 2008). The type of socio-material activities with the object seem to be associated with the peculiarity of the symptomatology of each child and not so much to her/his chronological age. At the same time, certain modalities in approaching the physical world, i.e., sensory-motor activities, persist also in the occurrence of a more theoretically abstract level of sociomaterial activities (canonical and socio-interactive). Although further research is required to confirm this hypothesis, our findings seem to support that the relationship between the child and the physical world is not exclusively shaped through the evolution of his/her cognitive understanding.

The results concerning the observations, in the different situations examined, seem to confirm the above mentioned insights showing the role played by the socio-material context in shaping the interpersonal relationship. Although the observations do not provide sufficient evidence to fully support the hypothesis of a full-fledged role of mediation of materiality in the psychological processes, it is still reasonable to support it, as already highlighted in the preliminary research (Iannaccone et al., 2018). We believe that these results deepen our knowledge of the “humility of things” (Miller, 2010), allowing us to address at least partially what Malafouris rightly claimed: “We constantly think through things, actively engaging our surrounding material environment, but we rarely become explicitly aware of the action potential of this engagement in the shaping of our minds and brains” (Malafouris, 2013, p.7).

## Conclusion, Limitations and Future Directions

This study highlights how the sociomaterial perspective provides important insights on how ASD children interact with the physical and social world. In particular, our findings show that children independent of age prefer sensory-motor activities with objects. These activities also seem to persist in children displaying more abstract-level activities, i.e., canonical and socio-interactive. Finally, the results show that objects allow children to shape the relationship with their partner and, even if a preliminary hypothesis, can mediate the relationships. Overall these results provide at least two important considerations for the interventions with children with ASD: the first concerns the analysis of children’s activities with objects considering the socio-material context of interaction, which could provide important information on children specific modalities of interacting with the physical and social world in different contexts, from the household to the therapy to the school; the second concerns the use of objects as mediators of the relationship between children and adults, specifically the objects could represent a starting point for establishing a communicative relationship based on the specific activities of the child. Finally, the analysis of children’s activities with objects in their socio-material context in interaction with a partner could retrospectively provide important information for the diagnosis.

The study presents some limitations: a non-homogeneous sample, the absence of a comparison sample of typically developing children and, finally, the absence of a peer partner. For these reasons, future studies should test a homogeneous sample concerning the severity of the symptomatology and the type of sociomaterial activities observed. In addition, it will be necessary to recruit a control group to confirm if the results of this study are specific for ASD children. Finally, to verify whether the children’s sociomaterial activities identified change as function of the type of the partner, it will be necessary to compare the same situations with a peer.

## Data Availability Statement

The datasets generated for this study are available on request to the corresponding author.

## Ethics Statement

This study was carried out in accordance with the recommendations of Associazione Italiana di Psicologia (AIP), and all of the children’s parents gave written informed consent in accordance with the Declaration of Helsinki. As there is no psychological ethics committee at the University of Salerno, the protocol was approved by an independent committee from the University’s Centro di Counseling Psicologico (Psychological Counseling Centre). This external committee supervises research carried out by psychological researchers at the university.

## Author Contributions

FM, GS, MM, and AI conceived and designed the experiment. FM, GS, and MM conducted the experiments in the centers. GS and MM secured ethical approval. FM, GS, and AI carried out the statistical and qualitative analyses. All authors contributed to the writing of the manuscript.

## Conflict of Interest

The authors declare that the research was conducted in the absence of any commercial or financial relationships that could be construed as a potential conflict of interest.

## Appendix

### Appendix 1: Socio-Material Use of Objects (SMUO check-list)

1. Is the child paying attention to the whole object, or to parts of it?
2. Is the child pointing at the object?
3. Has the child shared joint attention with the adult?
4. Does the child understand the use of the object?
5. For how many seconds does the child observe, indicate or touch the object?
6. Does the child imitate what the adult does with the object?
7. Is the child picking up the object as requested?
8. Does the child say the name of the object?
9. Does the child share the object with the adult?
10. Does the child use the object for its conventional purpose?
11. Does the child combine objects according to their conventional characteristics?
12. Does the child use the object to represent something else?
13. Does the child pretend to use an object that is present?
14. Does the child pretend to use an object that is not present?

## References

[B1] American Psychiatric Association [APA] (2013). *Diagnostic and Statistical Manual of Mental Disorders (DSM-5^∗∗^).* Washington, DC: Aufl. APA-Press.

[B2] Bachevalier J. (1994). Medial temporal lobe structures and autism: a review of clinical and experimental findings. *Neuropsychologia* 32 627–648. 10.1016/0028-3932(94)90025-6 8084420

[B3] Bakeman R. Adamson L. B. Konner M. Barr R. G. (1990). Kung infancy: the social context of object exploration. *Child Dev.* 61 794–809. 2364754

[B4] Barthélémy-Musso A. Tartas V. Guidetti M. (2013). Prendre les objets et leurs usages au sérieux: approche développementale de la co-construction de conventions sémiotiques entre enfants. *Psychol. Française* 58 67–88. 10.1016/j.psfr.2012.10.001

[B5] Ben-Ari R. Kedem-Friedrich P. (2000). Restructuring heterogeneous classes for cognitive development: social interactive perspective. *Instruct. Sci.* 28 153–167.

[B6] Bruckner C. T. Yoder P. (2007). Restricted object use in young children with autism: definition and construct validity. *Autism* 11 161–171. 10.1177/1362361307075709 17353216

[B7] Cattaruzza E. (2018). Exploring children’s agency in a designed atelier: a sociomaterial perspective. *Psihologija* 51 20–21.

[B8] Cattaruzza E. (2019). *A Sociomaterial Perspective for Learning: Exploring Atelier Activities.* Doctoral Thesis, Université de Neuchâtel, Neuchâtel.

[B9] Cattaruzza E. Iannaccone A. Arcidiacono F. (2019a). Provoking social changes in a family-school space of activity. *Psychol. Soc.* 11 33–47.

[B10] Cattaruzza E. Ligorio M. B. Iannaccone A. (2019b). Sociomateriality as a partner in the polyphony of students positioning. *Learn. Cult. Soc. Int.* 22:100332 10.1016/j.lcsi.2019.100332

[B11] Clark A. Chalmers D. (1998). The extended mind. *Analysis* 58 7–19.

[B12] Coppola C. Mollo M. Pacelli T. (2019). The worlds’ game: collective language manipulation as a space to develop logical abilities in a primary school classroom. *Eur. J. Psychol. Educ.* 34 783–799. 10.1007/s10212-018-0401-1

[B13] De Stefano C. T. Muller E. (1982). Environmental determinants of peer social activity in 18-month-old males. *Infant Behav. Dev.* 5 175–183. 10.1016/s0163-6383(82)80026-x

[B14] Di Dio C. Manzi F. Peretti G. Cangelosi A. Harris P. L. Massaro D. (2020a). Come i bambini pensano alla mente del robot: Il ruolo dell’attaccamento e della teoria della mente nell’attribuzione di stati mentali ad un agente robotico. *Sistemi Intell.* 1 41–56.

[B15] Di Dio C. Manzi F. Peretti G. Cangelosi A. Harris P. L. Massaro D. (2020b). Shall I trust you? From child human–robot interaction to trusting relationships. *Front. Psychol.* 11:469. 10.3389/fpsyg.2020.00469 32317998 PMC7147504

[B16] Di Dio C. Manzi F. Itakura S. Kanda T. Ishiguro H. Massaro D., et al. (2019). It does not matter who you are: fairness in pre-schoolers interacting with human and robotic partners. *Int. J. Soc. Robot.* 1–15.

[B17] Dimitrova N. Moro C. (2013). Common ground on object use associates with caregivers’ gestures. *Infant Behav. Dev.* 36 618–626. 10.1016/j.infbeh.2013.06.006 23891648

[B18] Dominguez A. Ziviani J. Rodger S. (2006). Play behaviours and play object preferences of young children with autistic disorder in a clinical play environment. *Autism* 10 53–69. 10.1177/1362361306062010 16522710

[B19] Iannaccone A. (2015). “Materiality and educational psychology. Paper Presented at the Symposium Materiality and Human Development,” in *16th Meeting of the International Society for Theoretical Psychology*, Coventry.

[B20] Iannaccone A. (2017). “Èduquerpeutêtredur! Quelques notes autour de la notion de matérialitéenéducation,” in *Lesinteractionssocialesenclasse: Réflexions et Perspectives*, eds Giglio M. Arcidiacono F. (Berne: Peter Lang).

[B21] Iannaccone A. Bruner J. S. (2010). *Le Condizioni Sociali Del Pensiero: Contesti, Attività e Ricerca di Senso.* Milano: Unicopli.

[B22] Iannaccone A. Perret-Clermont A. N. Convertini J. (2019). Children as investigators of Brunerian ‘possible worlds’. The role of narrative scenarios in children’s argumentative thinking. *Integrat. Psychol. Behav. Sci.* 53 679–693. 10.1007/s12124-019-09505-3 31729627

[B23] Iannaccone A. Savarese G. Manzi F. (2016). “The use of objects for autistic children: a study in Piagetian perspective and the use of construction blocks,” in *Poster presented at the Conference: ‘XXIX Congresso Nazionale AIP – Sezione di PsicologiadelloSviluppo e dell’Educazione’*, Vicenza.

[B24] Iannaccone A. Savarese G. Manzi F. (2018). Object use in children with autism: building with blocks from a Piagetian perspective. *Front. Educ.* 3:12 10.3389/feduc.2018.00012

[B25] Jacobson J. L. (1981). The role of inanimate objects in early peer interaction. *Child Dev.* 52 618–626.

[B26] Jarrold C. (2003). A review of research into pretend play in autism. *Autism* 7 379–390. 10.1177/1362361303007004004 14678677

[B27] Jarrold C. Boucher J. Smith P. (1993). Symbolic play in autism: a review. *J. Autism. Dev. Disord.* 23 281–307. 10.1007/bf01046221 7687245

[B28] Kanner L. (1943). Autistic disturbances of affective contact. *Nervous Child* 2 217–250. 4880460

[B29] Lieber J. Beckman P. J. (1991). The role of toys in individual and dyadic play among young children with handicaps. *J. Appl. Dev. Psychol.* 12 189–203. 10.1016/0193-3973(91)90011-r

[B30] Ligorio M. B. (2010). Dialogical relationship between identity and learning. *Cult. Psychol.* 16 93–107. 10.1177/1354067x09353206

[B31] Linell P. (2009). *Rethinking Language, Mind, and World Dialogically: Interactional and Contextual Theories of Human Sense-Making.* Charlotte, NC: Information Age Publishing, Inc.

[B32] Lord C. (1983). “Autism and the comprehension of language,” in *Communication Problems in Autism*, eds Schopler E. Mesibov G. B. (New York, NY: Plenum Press), 257–281. 10.1007/978-1-4757-4806-2_14

[B33] Lord C. (1984). “The development of peer relations in children with autism,” in *Advances in Applied Developmental Psychology*, eds Morrison F. J. Lord C. Keating D. P. (New York, NY: Harcourt), 165–227.

[B34] Lord C. Hopkins J. M. (1986). The social behaviour of autistic children with younger and same-age nonhandicapped peers. *J. Autism. Dev. Disord.* 16 249–262. 10.1007/bf01531658 3558287

[B35] Loveland K. A. Tunali B. (1991). Social scripts for conversational interactions in autism and Down syndrome. *J. Autism. Dev. Disord.* 21 177–186. 10.1007/bf02284758 1830877

[B36] Malafouris L. (2013). *How Things Shape the Mind: A Theory of Material Engagement.* Cambridge: MIT Press.

[B37] Malafouris L. (2019). Understanding the effects of materiality on mental health. *Br. J. Psych. Bull.* 43 195–200. 10.1192/bjb.2019.7 30837025 PMC12402920

[B38] Manzi F. (2018). *Materiality and the Construction of Intersubjectivity: Human–Robot Interaction in Typical Development and the use of the Objects in ASD Children.* Doctoral Thesis, Università Cattolica del Sacro Cuore di Milano, Milano, MI.

[B73] Manzi F. Di Dio C. Itakura S. Kanda T. Ishiguro H. Massaro D. (2020). *Moral evaluation of Human and Robot interactions in Japanese preschoolers*. Italy: CEUR Workshop Proceedings Conference.

[B39] Manzi F. Massaro D. Kanda T. Tomita K. Itakura S. Marchetti A. (2017). “Teoria della Mente, bambini e robot: l’attribuzione di stati mentali,” in *Paper presented at XXX Congresso AIP Sezione di Psicologia dello Sviluppo e dell’Educazione*, Messina.

[B40] Manzi F. Savarese G. (2017). “Interaction with/through object and social functions in ASD children,” in *Abstract Book of the International Congress: Educa 2017 ‘Inequalities: What Contributions of the Educations for … ?*, Hammamet, 73.

[B41] Marchetti A. Manzi F. Itakura S. Massaro D. (2018). Theory of mind and humanoid robots from a lifespan perspective. *Z. Psychol.* 226 98–109. 10.1027/2151-2604/a000326

[B42] Marchetti A. Miraglia L. Di Dio C. (2020). Towards a socio-material approach to cognitive empathy in autistic spectrum disorder. *Front. Psychol.* 10:02965. 10.3389/fpsyg.2019.02965 31998198 PMC6967260

[B43] Miller D. (2010). *Stuff.* Cambridge: Polity.

[B44] Moreno-Núñez A. Rodríguez C. Del Olmo M. J. (2017). Rhythmic ostensive gestures: How adults facilitate infants’ entrance into early triadic interactions. *Infant Behav. Dev.* 49 168–181. 10.1016/j.infbeh.2017.09.003 28946022

[B45] Moro C. (2011). “Material culture, semiotics and early childhood development,” in *Children, Development and Education: Cultural, Historical, Anthropological Perspectives*, eds Kontopodis M. Wulf C. Fichtner B. (New York, NY: Springer Verlag), 57–70. 10.1007/978-94-007-0243-1_4

[B46] Moro C. (2014). “Le rôle de l’objetdans la construction de l’attention con jointe et dansl’accès aux intentions d’autrui,” in *Sémiotique, Culture et Développement Psychologique*, eds Moro C. Muller Mirza N. (Lille, FR: Presses Universitaires du Septentrion), 55–77.

[B47] Moscovici S. (1976). *La Psychanalyse: Son Image et Son Public [Psychoanalysis: Its image and its public].* Oxford: U France Press.

[B48] Mottron L. Mineau S. Martel G. Bernier C. S. C. Berthiaume C. Dawson M. (2007). Lateral glances toward moving stimuli among young children with autism: early regulation of locally oriented perception? *Dev. Psychopathol.* 19 23–36. 17241482 10.1017/S0954579407070022

[B49] Ozonoff S. Macari S. Young G. S. Goldring S. Thompson M. Rogers S. J. (2008). Atypical object exploration at 12 months of age is associated with autism in a prospective sample. *Autism* 12 457–472. 10.1177/1362361308096402 18805942 PMC2921192

[B50] Perret-Clermont A.-N. (2004). “The thinking spaces of the young,” in *Joining Society: Social Interactions and Learning in Adolescence and Youth*, eds Perret-Clermont A.-N. Pontecorvo C. Resnick L. Zittoun T. Burge B. (New York, NY: Cambridge University Press), 3–10. 10.1017/cbo9780511616341.003

[B51] Piaget J. (1928). *The child’s Conception of the World.* London, UK: Routledge and Kegan Paul.

[B52] Piaget J. (1952a). *Play, Dreams and Imitation in Childhood.* New York, NY: Norton.

[B53] Piaget J. (1952b). *The origins of Intelligence in Children.* New York, NY: Norton.

[B54] Piaget J. (1954). *The Construction of Reality in the Child.* New York, NY: Basic Books.

[B55] Piaget J. (1962). *Play, Dreams and Imitation in Childhood.* New York, NY: Norton.

[B56] Piaget J. (1972). *The Psychology of the Child.* New York, NY: Basic Books.

[B57] Radford L. (2003). On the epistemological limits of language: mathematical knowledge and social practice during the Renaissance. *Educ. Stud. Math.* 52 123–150.

[B58] Rodríguez C. Moreno-Núñez A. Basilio M. Sosa N. (2015). Ostensive gestures come first: their role in the beginning of shared reference. *Cogn. Dev.* 36 142–149. 10.1016/j.cogdev.2015.09.005

[B59] Rodríguez C. Moro C. (1998). El usoconvencionaltambiénhacepermanentes a los objetos. *Infancia y Aprendizaje* 21 67–83.

[B60] Romanczyk R. G. Goren E. R. (1975). Severe self-injurious behavior: the problem of clinical control. *J. Consult. Clin. Psychol.* 43 730–739. 10.1037/0022-006x.43.5.730 1176689

[B61] Savarese G. Manzi F. Iannaccone A. (2017). Social functions in ASD children and interaction with/through object: a brief report. *Psychology* 8 1129–1133. 10.4236/psych.2017.88073

[B62] Sigman M. Ungerer J. A. (1984). Cognitive and language skills in autistic, mentally retarded, and normal children. *Dev. Psychol.* 20 293–302. 10.1037/0012-1649.20.2.293

[B63] Tomasello M. (2016). Cultural learning redux. *Child Dev.* 87 643–653. 10.1111/cdev.12499 27189393

[B64] Tomasello M. Conti-Ramsden G. Ewert B. (1990). Young children’s conversations with their mothers and fathers: differences in breakdown and repair. *J. Child Lang.* 17 115–130. 10.1017/s0305000900013131 2312636

[B65] Trevarthen C. Hubley P. (1978). “Secondary intersubjectivity: confidence, confiding, and acts of meaning in the first year,” in *Action, Gesture, and Symbol*, ed. Lock J. (London: Academic Press), 183–229.

[B66] Turkle S. (2004). Whither psychoanalysis in computer culture? *Psychoanal. Psychol.* 21 16–30. 10.1037/0736-9735.21.1.16

[B67] Vygotskij L. S. (1976). *Immaginazione e Creatività Infantile.* Roma: Editori Riuniti.

[B68] Vygotsky L. S. (2004). Imagination and creativity in childhood. *J. Russ. East Eur. Psychol.* 42 7–97.

[B69] Williams E. Costall A. Reddy V. (1999). Children with autism experience problems with both objects and people. *J. Autism. Dev. Disord.* 29 367–378. 10587883 10.1023/a:1023026810619

[B70] Williams E. Kendell-Scott L. Costall A. (2005). Parents’ experiences of introducing everyday object use to their children with autism. *Autism* 9 495–514. 10.1177/1362361305057869 16287702

[B71] Ziviani J. Boyle M. Rodger S. (2001). An introduction to play and the preschool child with autistic spectrum disorder. *Br. J. Occup. Ther.* 64 17–22. 10.1177/030802260106400104

[B72] Zucchermaglio C. (1996). *Vygotskij in Azienda. Apprendimento e Comunicazione nei Contesti Lavorativi.* Roma: Carocci.

